# Tensor-valued diffusion encoding for diffusional variance decomposition (DIVIDE): Technical feasibility in clinical MRI systems

**DOI:** 10.1371/journal.pone.0214238

**Published:** 2019-03-28

**Authors:** Filip Szczepankiewicz, Jens Sjölund, Freddy Ståhlberg, Jimmy Lätt, Markus Nilsson

**Affiliations:** 1 Lund University, Department of Clinical Sciences Lund, Medical Radiation Physics, Lund, Sweden; 2 Elekta Instrument AB, Kungstensgatan 18, Stockholm, Sweden; 3 Linköping University, Department of Biomedical Engineering, Linköping, Sweden; 4 Linköping University, Center for Medical Image Science and Visualization (CMIV), Linköping, Sweden; 5 Lund University, Department of Clinical Sciences Lund, Diagnostic Radiology, Lund, Sweden; 6 Skåne University Hospital, Department of Imaging and Function, Lund, Sweden; 7 Lund University, Lund University Bioimaging Center, Lund, Sweden; McLean Hospital, UNITED STATES

## Abstract

Microstructure imaging techniques based on tensor-valued diffusion encoding have gained popularity within the MRI research community. Unlike conventional diffusion encoding—applied along a single direction in each shot—tensor-valued encoding employs diffusion encoding along multiple directions within a single preparation of the signal. The benefit is that such encoding may probe tissue features that are not accessible by conventional encoding. For example, diffusional variance decomposition (DIVIDE) takes advantage of tensor-valued encoding to probe microscopic diffusion anisotropy independent of orientation coherence. The drawback is that tensor-valued encoding generally requires gradient waveforms that are more demanding on hardware; it has therefore been used primarily in MRI systems with relatively high performance. The purpose of this work was to explore tensor-valued diffusion encoding on clinical MRI systems with varying performance to test its technical feasibility within the context of DIVIDE. We performed whole-brain imaging with linear and spherical b-tensor encoding at field strengths between 1.5 and 7 T, and at maximal gradient amplitudes between 45 and 80 mT/m. Asymmetric gradient waveforms were optimized numerically to yield b-values up to 2 ms/μm^2^. Technical feasibility was assessed in terms of the repeatability, SNR, and quality of DIVIDE parameter maps. Variable system performance resulted in echo times between 83 to 115 ms and total acquisition times of 6 to 9 minutes when using 80 signal samples and resolution 2×2×4 mm^3^. As expected, the repeatability, signal-to-noise ratio and parameter map quality depended on hardware performance. We conclude that tensor-valued encoding is feasible for a wide range of MRI systems—even at 1.5 T with maximal gradient waveform amplitudes of 33 mT/m—and baseline experimental design and quality parameters for all included configurations. This demonstrates that tissue features, beyond those accessible by conventional diffusion encoding, can be explored on a wide range of MRI systems.

## Introduction

Diffusion magnetic resonance imaging (dMRI) enables non-invasive imaging of tissue microstructure. The vast majority of dMRI techniques rely on the conventional Stejskal-Tanner experiment [1] that employs a pair of pulsed field gradients to yield diffusion encoding along a single direction for each preparation of the signal, so called ‘linear’ encoding. Methods such as diffusion tensor imaging (DTI) [2] yield voxel-scale average parameters which are sensitive to alterations of the tissue microstructure in both healthy development and disease [3]. Diffusional kurtosis imaging (DKI) [4] is an extension to DTI that can probe non-gaussian processes and sub-voxel heterogeneity—a feature that has the potential to differentiate, for example, high and low-grade gliomas [5, 6]. Information on the microstructure can also be inferred from biophysical models, which may provide more specific information based on assumptions about the tissue composition [7]. However, techniques that rely on linear diffusion encoding alone all have a fundamental drawback: they convolve the effects of microscopic diffusion anisotropy, orientation dispersion, and heterogeneous isotropic diffusivity [8–11]. Consequently, markedly different tissue archetypes, such as elongated cell structures that are randomly oriented and isotropic tissues with varying cell density, may be indistinguishable regardless of the modelling approach [8, 9].

This limitation can be mitigated by performing diffusion encoding along multiple directions in a single acquisition. This approach was introduced by Cory et al. [12], who proposed parallel and orthogonal double diffusion encoding (DDE) as a probe of local pore geometry. This concept has since been elaborated on by many contributors [13]. Mori and van Zijl [14] and Wong et al. [15] introduced isotropic diffusion encoding by pulsed field gradients in order to sensitize the signal to the direction-average diffusivity without the need for multiple encoding directions, which may be useful in rapid diffusion-weighted imaging [16, 17]. Eriksson et al. [18] later demonstrated that a combination of linear and isotropic encoding is sensitive to microscopic anisotropy [19] and that isotropic encoding can be obtained by continuously varying the gradient waveform, a premise that facilitates waveform optimization based on arbitrary gradient trajectories [20].

To emphasize that the encoding is no longer described by a vector (a single gradient direction), we refer to it as *tensor-valued diffusion encoding* and describe it by a b-tensor. The tensor can be characterized by its trace (b-value) and its symmetry axis (encoding direction). It can also be assigned a *shape* defined from the b-tensor eigenvalues, similar to shape parameters related to the diffusion tensor [21, 22]. The b-tensor formalism was proposed by Westin et al. [21] and generalizes to arbitrary gradient trajectories. Variation of the b-tensor shape is central to probing microscopic anisotropy, as demonstrated for angular DDE that yields linear and planar tensor encoding (LTE and PTE) [12, 13, 23–28], and for combinations of LTE and spherical tensor encoding (STE) [9, 19].

Acquisition based on gradient waveforms that yield multiple shapes of the b-tensor can be analyzed in a variety of ways [19, 21–24, 29–31]. In this work we will use so-called diffusional variance decomposition (DIVIDE) [19, 21, 32] which is a simple signal representation that captures the mean diffusivity and diffusional variance (or kurtosis) caused by microscopic anisotropy and heterogeneous isotropic diffusivity [9, 19].

The gradient waveforms necessary to yield, for example, planar or spherical b-tensors, are generally more demanding on the gradient hardware compared to encoding along a single direction. As such, these techniques have primarily been used with high-performance gradient systems that facilitate sufficient data quality and acceptable acquisition times. For example, an early clinical study where microscopic anisotropy was investigated based on a combination of linear and spherical encoding was performed at a 3 T scanner with 80 mT/m gradients—currently considered a high-performance system. Even so, it required an echo time of 160 ms to achieve a b-value of 2.8 ms/μm^2^ which limited the spatial resolution and coverage [32]. This warrants development of efficient gradient waveform design [20], parsimonious signal sampling protocols, and investigations of the technical feasibility in MRI systems with different performance.

In this study we aim to survey the technical feasibility of tensor-valued diffusion encoding for DIVIDE analysis, specifically linear and spherical b-tensors, adapted for a whole-brain acquisitions over a wide range of MRI systems. We consider four configurations with field strengths between 1.5 and 7 T, and maximum gradient amplitudes between 45 and 80 mT/m. The most challenging configurations were expected to be those where the SNR was low due to strong T2 relaxation, i.e. configurations where a low maximal gradient amplitudes led to long echo times or with ultra-high field strengths that led to shorter transversal relaxation times [33–35].

## Methods

We investigated the technical feasibility of tensor-valued diffusion encoding on three MRI scanners with field strengths between 1.5 and 7 T, and maximal gradient amplitudes between 45 and 80 mT/m. The three scanners were used in four configurations (A-D) as described in Table 1. Notably, configurations B and C use the same MR scanner, but configuration B used the protocol and waveforms optimized for configuration A. Furthermore, we emphasize that configuration D employed experimental hardware in a clinical setting.

**Table 1:** Configuration of hardware, imaging protocols and gradient waveforms. Configurations A-C used 20-channel receive head/neck coil arrays. Configuration D used a 32-channel transmit/receive head coil array. The maximal gradient waveform amplitude and slew rate allowed by the hardware (*G*_max_ and *S*_max_) and the maximum that was used for STE and LTE are stated along with imaging and waveform timing parameters.

	*Unit*	A	B	C	D	
Scanner		I	II	II	III	
B_0_	T	1.5	3	3	7	
*G*_max_	mT/m	45	80	80	60	
*S*_max_	T/m/s	200	200	200	100	
					
*Imaging parameters*					
TE	ms	115	115	83	88	
TR	ms	5000	5000	4100	6500	
*T*_tot_	min	6:40	6:40	5:28	8:40	
					
*Waveform parameters*					
*G*_STE_, *G*_LTE_	mT/m	33, 33	33, 33	66, 67	55, 60	
*S*_STE_, *S*_LTE_	T/m/s	25, 13	25, 18	64, 59	58, 40	
STE δ_1_, δ_2_, δ_P_	ms	49, 42, 7	49, 42, 8	32, 26, 8	36, 27, 7	
LTE δ_1_, δ_2_, δ_P_	ms	42, 42, 7	42, 42, 8	26, 26, 8	27, 27, 16	
					

Scanner I: Siemens Magnetom Aera; II: Siemens Magnetom Prisma; III: Philips Achieva 7T. B_0_ main magnetic field strength; *G*_max_ maximum gradient amplitude; *S*_max_ maximum gradient slew rate; TE echo time; TR repetition time; *T*_tot_ total acquisition time excluding preparation stages; *G*_STE_, *G*_LTE_ and *S*_STE_, *S*_LTE_ are the maximal amplitudes and slew rates used in the STE and LTE waveforms; δ_1_ and δ_2_ duration of waveform before and after refocusing pulse; δ_P_ is the time between the end of the first and beginning of the second waveform.

All configurations were investigated using the clinically relevant requirements of whole-brain coverage (120 mm contiguous coverage in feet-head direction) and acquisition times below 10 minutes. Technical feasibility was quantified in terms of the SNR across the brain parenchyma, and the repeatability of the diffusion-weighted signal attenuation and the estimated DIVIDE parameters. Furthermore, qualitative features such as image artifacts, were noted.

### Protocol design and data acquisition

The protocols were designed using the following three steps: (i) set the minimal and maximal b-values and number of b-values, (ii) determine the minimal echo and repetition times (TE and TR) for each configuration, (iii) distribute the available signal samples across b-values, encoding directions and b-tensor shapes. Details for each step are given in the Supporting Information. Protocols and waveforms were separately optimized for each configuration and are summarized in Table 1. The following imaging parameters were kept constant for all configurations: acquisition matrix 112×112 in 30 contiguous slices, partial-Fourier 0.75, in-plane acceleration factor 2 (A-C: GRAPPA, D: SENSE), bandwidth 1800 Hz/pixel, and fat suppression was engaged (A-C: ‘strong’, D: ‘strong’ with slice gradient reversal). Notably, the default spatial resolution was 2×2×4 mm^3^; the effect of anisotropic voxels is commented on in the discussion. The strength of the diffusion encoding was *b* = 0.1, 0.7, 1.4 and 2 ms/μm^2^ for both LTE and STE. For these b-values, the number of directions for LTE was 6, 6, 12, and 16, respectively. The STE was not rotated since it is assumed to be rotation invariant, but was repeated 6, 6, 12, and 16 times, respectively. This resulted in a total of 80 signal samples per voxel. The sampling order was volume-interleaved to distribute energy consumption and heating more evenly over time [36] and to reduce the potential bias caused by system drift [37]. The total acquisition time (*T*_tot_) was calculated as the number of samples multiplied by TR to remove dependency on preparation phases that may be different across configurations.

**Fig 1:**
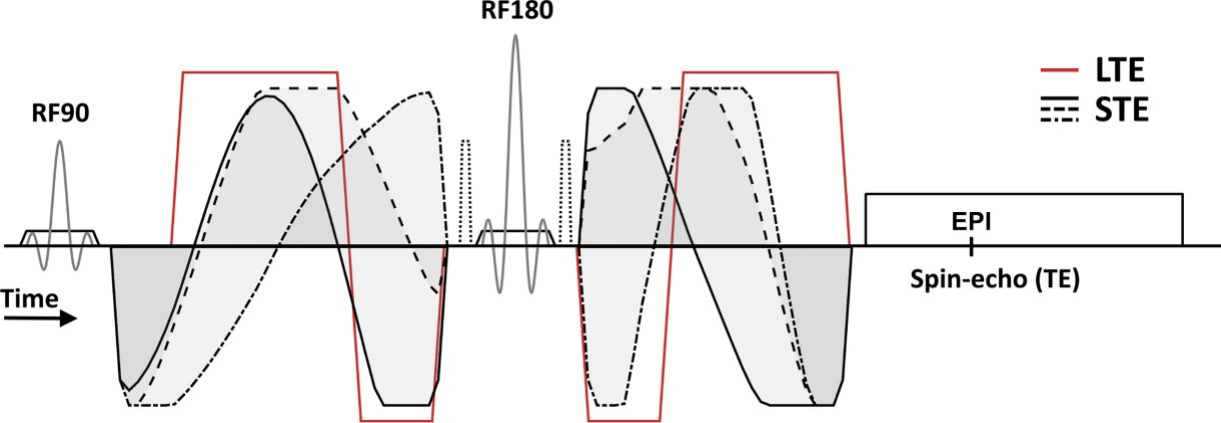
**Schematic spin-echo sequence with echo planar imaging readout and diffusion-encoding gradient waveforms that yield linear (red line) and spherical b-tensor encoding (black lines).** Note that the STE waveform is asymmetric around the refocusing pulse [20] and that it must therefore be designed to compensate for errors caused by concomitant fields [40]. The LTE waveform is bipolar to match the effective diffusion times for STE and LTE [41]. Note that crusher gradients (dotted lines) are not engaged when the diffusion encoding acts as a crusher.

All experiments were performed using an in-house developed prototype pulse sequence based on the diffusion-weighted spin-echo sequence with echo-planar imaging readout provided by Philips Healthcare (Best, the Netherlands) and Siemens Healthcare (Erlangen, Germany). Asymmetric gradient waveforms for STE were optimized for each configuration to minimize TE using the software described by Sjölund et al. [20], available at https://github.com/jsjol/NOW. The optimization of gradient waveforms used the max norm, heat dissipation factor 0.6, and a slew rate limit of 100 T/m/s, to comply with both duty cycle and peripheral nerve stimulation limits [38, 39]. The actual slew rate was determined by the temporal resolution of the waveform optimization as well as the amplitude and timing of the waveform. Although this imposed relatively low slew rates compared to the limit used in optimization, it is expected to have marginal effect on encoding efficiency. As noted in Table 1, the STE gradient amplitude was not maximized because such waveforms could exceed the duty cycle of the scanner. Instead, several amplitudes were tested empirically, and the most efficient waveform that was robustly executed was selected. The waveform optimization included concomitant field effect compensation, and all waveforms had Maxwell indices below 200 (mT/m)^2^ms which ensures negligible signal error [40]. The LTE used symmetric bipolar trapezoid waveforms to approximately match the gradient amplitude and diffusion time of the STE experiment [41]. An example of a spin-echo sequence with both types of waveforms is shown in Fig 1. Comprehensive definitions of waveforms, timing settings, signal sampling schemes and model fit settings are available at https://github.com/filip-szczepankiewicz/Szczepankiewicz_PONE_2019.

All data were acquired twice within the same session for each configuration, in a single healthy volunteer (male, age 27 y) with previous experience as an MRI volunteer. There was no compensation for volunteering. All experiments were approved by the Regional Ethical Review Board (Lund, Sweden, EPN 2014–735 for systems I and II, EPN 2012–428 for system III), and informed consent was obtained prior to participation. Diffusion-weighted data were corrected for motion and eddy currents in Elastix [42] using an extrapolation-based reference volume [43]. To retain the impact of signal noise on the images, no smoothing was applied at any stage of the processing.

To investigate the interplay between SNR and spatial resolution, we created two alternative configurations. Based on configuration A, we mitigated the low SNR by reducing the spatial resolution. A low-resolution configuration (A*) was achieved by setting the matrix size to 88×88 resulting in 2.5×2.5×4 mm^3^ voxels and the bandwidth was reduced to 1180 Hz/pixel to preserve the readout time and total acquisition time. Based on configuration C, we sacrificed SNR in favor of an increased spatial resolution. A high-resolution configuration (C*) was achieved by reducing the slice thickness to 2 mm. To preserve coverage, the number of slices was doubled and the repetition time was set to 8900 ms. This yielded a spatial resolution of 2×2×2 mm^3^ and an acquisition time of 12 minutes.

### DIVIDE parameter estimation

DIVIDE employs a joint analysis of data acquired with b-tensors (**B**) with multiple b-values (*b* = Trace(**B**)) and shapes (*b*_Δ_) [22]. Assuming that the observed diffusion process can be approximated by a distribution of diffusion tensors (multi-Gaussian) [4, 44], the fitting parameters can be interpreted in terms of the signal at *b* = 0 (*S*_0_), mean diffusivity (MD) and the anisotropic and isotropic diffusional variance (*V*_A_ and *V*_I_) [19, 32]. Although several approaches for estimation of these parameters exist [21, 24, 29, 45], we estimate the parameters by fitting the Laplace transform of the gamma distribution [46, 47] to the powder-averaged LTE and STE signal ($\bar{S}$) simultaneously [19, 32], according to

$$\bar{S}(b,b_{\Delta })=S_{0}{\left(1+b\cdot \frac{V_{I}+b_{\Delta }^{2}\cdot V_{A}}{MD}\right)}^{-\frac{{MD}^{2}}{V_{I}+b_{\Delta }^{2}\cdot V_{A}}}.$$ Eq 1

For LTE, the powder average is the arithmetic average across directions for each b-value (see Supporting Information), whereas STE is natively rotation invariant and requires no such averaging. Note that Eq 1 is only valid for axisymmetric b-tensors, and that *b*_Δ_ is 1 and 0 for LTE and STE, respectively [48]. The average and variance of the gamma distribution produced by the fit are estimates of the average and variance of the true distribution of diffusion coefficients. If the gamma and true distributions have different functional forms, the estimates may be biased. The fitting was initialized at random values, accounted for heteroscedasticity (weighted by the square root of the number of signal samples that make up each average), and was constrained to yield real—but not necessarily positive—values for *V*_I_ or *V*_A_. Negative variances are not physical but can be caused by noise and were allowed to avoid a positive bias for values close to zero. The diffusional variance was reported in terms of a normalized metric similar to the mean kurtosis from DKI [4], defined according to [19, 49]

$$MK_{x}=3\cdot \frac{V_{x}}{MD^{2}},$$ Eq 2

where ‘x’ denotes the isotropic (I) or anisotropic (A) diffusional variance. The normalized signal at *b* = 2 ms/μm^2^ averaged over directions or repetitions ($\bar{S}/S_{0}$) was also considered in the analysis of repeatability, and denoted *A*(L) and *A*(S) when using LTE and STE, respectively. Finally, we calculated the microscopic fractional anisotropy (μFA), according to

$$\mu FA=\sqrt{\frac{3}{2}}\cdot {\left(1+\frac{MD^{2}+V_{I}}{\frac{5}{2}V_{A}}\right)}^{-1/2},$$ Eq 3

which can be interpreted as the FA that would be observed if all structures in the sample were aligned in parallel [9, 21]. Thus, μFA captures the microscopic diffusional anisotropy even in samples that are isotropic on the voxel scale [24, 32, 50]. We use the definition by Westin et al. [21] (including the contribution from *V*_I_) rather than the one by Lasič et al. [19]. The post-processing and analysis software [51] was written in Matlab (The MathWorks, Natick, MA, USA) and is available at https://github.com/markus-nilsson/md-dmri.

### Analysis of repeatability

For each configuration, data from two identical acquisitions were compared to investigate repeatability. Spatial correspondence across acquisitions was achieved by not repositioning the subject between acquisitions. To gauge the signal and DIVIDE parameter repeatability, we calculated the differences between the first and second acquisition (Δ*X* = *X*_1_ –*X*_2_) [52], such that Δ*X* is a distribution of voxel-wise differences. The repeatability was visualized in maps of Δ*X* as well as Bland-Altman plots. The repeatability was summarized by the mean and standard deviation of Δ*X* to capture the overall parameter bias and precision. To avoid inflated variability due to a misaligned brain periphery and partial-volume effects with CSF, only voxels where μFA > 0.7 and MD < 1.5 μm^2^/ms were considered.

### Analysis of SNR

At low SNR, the MR signal is positively biased due to the rectified noise floor, with detrimental effects on parameter accuracy [53–55]. Since STE was repeated several times for each b-value, we could estimate the SNR at each b-value by computing the ratio between the mean of the STE signal and its standard deviation. By assuming that signal is approximately Rice distributed, we can use a threshold of SNR < 3 to mark regions where signal bias is likely to influence signal accuracy [56]. Doing so may identify regions of the brain that require action to improve SNR. To avoid overestimation of the SNR, it was calculated based on image data that had not been motion corrected. As a summary parameter of data quality, we report the fraction of the brain parenchyma where SNR was above 3 and 6 at the highest b-value (*b* = 2 ms/μm^2^), denoted *Q*_3_ and *Q*_6_. Only voxels where MD < 1.5 μm^2^/ms were included in the analysis to avoid regions dominated by cerebrospinal fluid.

### Simulation of parameter accuracy and precision

Simulations were used to investigate the impact of signal noise on the bias, variability and negative values of DIVIDE parameters. To this end, we used toy models of tissue described by two diffusion tensors (**D**), such that *S*(**B**)/*S*_0_ = *f* exp(−**B**:**D**_1_)+(1−*f*)exp(−**B**:**D**_2_). Note that this construct is only intended to mimic the signal and noise characteristics in brain and makes no claims about the actual brain microstructure. The first tissue model mimics white matter (high MK_A_ and low MK_I_), by using diffusion tensors with eigenvalues λ_**D**1_ = [2.1 0 0] μm^2^/ms, λ_**D**2_ = [1.4 1.4 1.4] μm^2^/ms and *f* = 0.8. The second model mimics isotropic tissue with high MK_I_ by using λ_**D**1_ = [0.4 0.4 0.4] μm^2^/ms, λ_**D**2_ = [1.4 1.4 1.4] μm^2^/ms and *f* = 0.5. Such values were uncommon in the healthy brain but may appear in tumor tissue [9]. Furthermore, we investigated if the positive MK_I_ values observed in vivo reflect the true presence of heterogeneous isotropic diffusivity or if this feature can be attributed to noise. The third tissue model therefore includes anisotropic and isotropic diffusion tensors with equal average diffusivity where λ_**D**1_ = [2.1 0 0] μm^2^/ms, λ_**D**2_ = [0.7 0.7 0.7] μm^2^/ms and *f* = 0.5. Since the true MK_I_ in this case is zero, this example visualizes the magnitude of the MK_I_ bias caused by noise as a function of SNR.

Noise sampled from a Rice distribution was added to yield SNR between 10 and 100 in *S*_0_. Each simulation used the same signal sampling scheme and fitting procedure as for the in vivo case. Simulations were repeated 10^4^ times using independent realizations of noise for each SNR value. The mean and standard deviation of model parameters across realizations was calculated to quantify their accuracy and precision.

## Results

**Fig 2:**
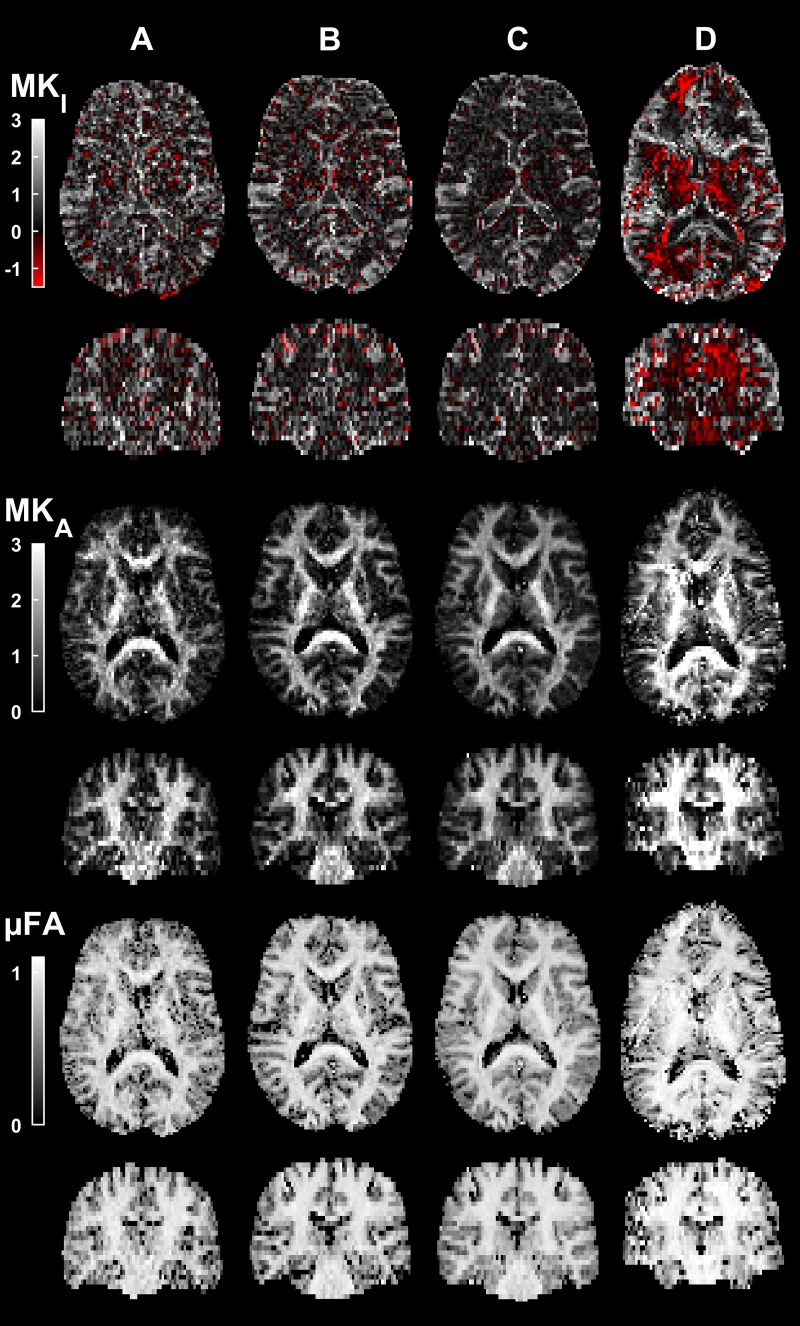
**DIVIDE parameter maps in transversal and coronal slices for configurations A-D.** The image quality generally improves with higher field strength and gradient amplitude. Most notably there is a discernably higher level of noise for configuration A. Configuration D generally exhibited higher MK_A_, a pronounced geometrical distortion at the anterior part of the brain, and more pronounced ghosting artifacts, as compared with the other configurations. Furthermore, large regions of negative MK_I_ were observed only for configuration D.

Tensor-valued diffusion encoding was successfully performed using all four configurations. Fig 2 shows the calculated DIVIDE parameter maps. There is an appreciable difference in image quality depending on hardware performance. As expected, higher field strengths and stronger gradients generally render lower echo times and higher image quality. The influence of noise was most prominent for configuration A. This is likely due to the long echo time (TE = 115 ms) incurred by the relatively low gradient amplitude. By contrast, SNR was relatively high for configuration D, but its parameter maps exhibited contrast that differed from other configurations. For example, MK_I_ was negative in large regions throughout the parenchyma, and MK_A_ was higher than at other systems, most prominently in coherent white matter pathways. Image artifacts were also noticeable, such as geometric distortions, likely associated to issues commonly observed for dMRI at ultra-high fields [57].

**Fig 3:**
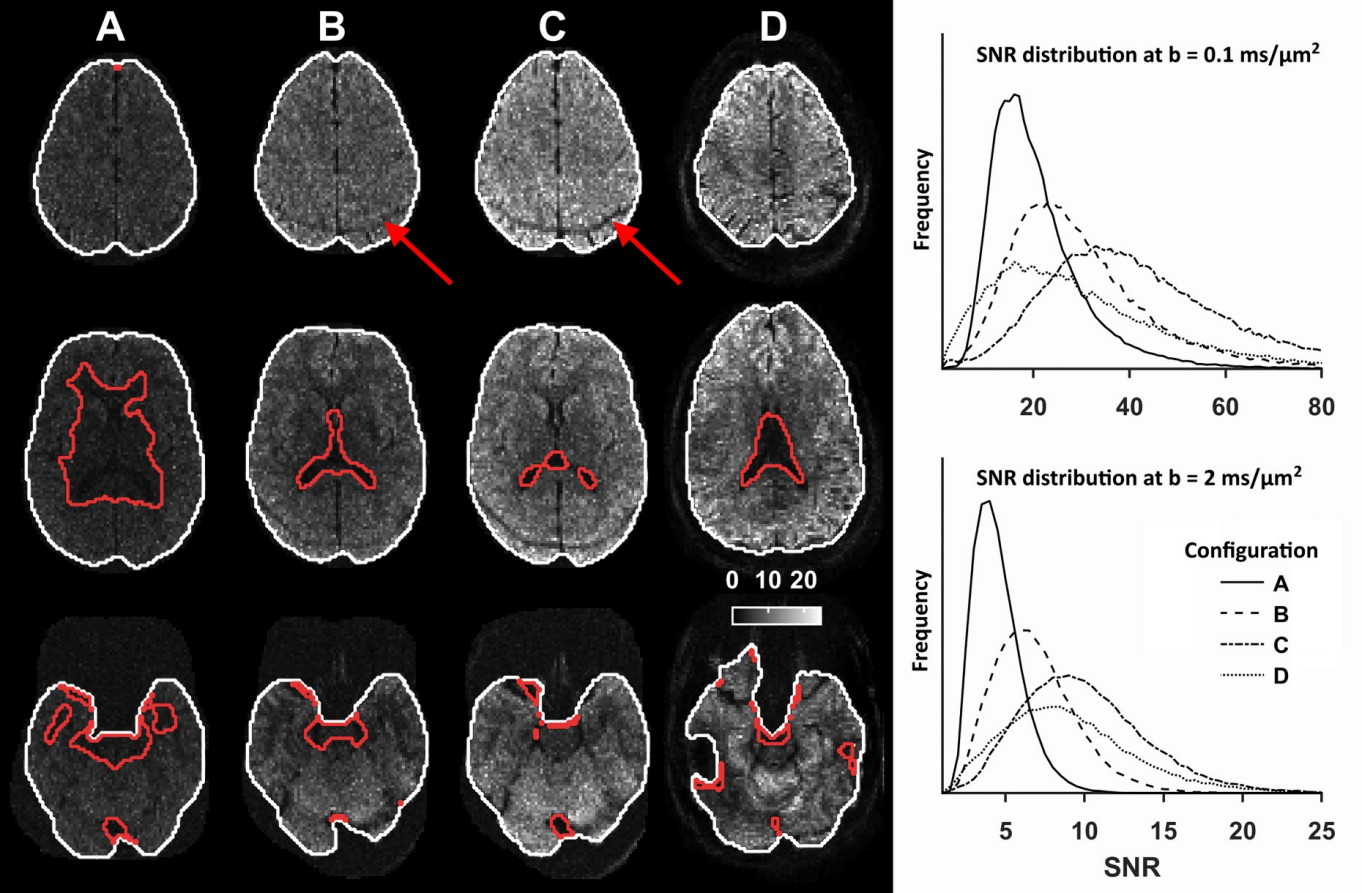
**SNR maps at *b* = 2 ms/**μ**m**^**2**^ **in three transversal slices for configurations A-D and SNR distributions in the brain parenchyma at *b* = 0.1 and 2 ms/μm^2^ (histograms).** White outlines show the outer perimeter within which SNR > 3, and the red outlines show regions where SNR < 3. At high b-values, images from all systems exhibited low SNR in the ventricles due to the high diffusivity of CSF. Configuration A shows low SNR in the central and inferior parts of the brain. Configuration C exhibited the highest SNR across all b-values, but also a posterior rim of low SNR caused by poor fat suppression (red arrows). Configuration D generally exhibited high SNR in the peripheral regions; in the lower parts of the brain, it exhibited a heterogeneous SNR. It also exhibited an irregular perimeter in the inferior part of the brain, which was likely caused by local field heterogeneity in proximity to the ear canal and poor RF homogeneity, both commonly associated to dMRI at ultra-high field strengths [58].

Fig 3 shows maps of the SNR at the highest b-value (*b* = 2 ms/μm^2^) in three axial slices for all configurations. Configuration A exhibited SNR < 3 close to the lateral ventricles and in the inferior parts of the brain. Configurations B-D exhibited SNR > 3 in more than 96% of the brain parenchyma. A complete list of the quality parameters is reported in Table 2.

**Table 2:** Quality and repeatability parameters for configurations A-D. *Q*_3_ and *Q*_6_ are the fraction of tissue where SNR is above 3 and 6 at *b* = 2 ms/μm^2^. The voxel-wise parameter difference between acquisition 1 and 2 (Δ*X*) is reported as the average ± one standard deviation. The lowest and highest SNR was observed for configurations A and C, respectively, and parameter precision follows the same pattern. Neither the signal attenuation nor DIVIDE parameters had a relevant bias compared to their standard deviation. Furthermore, MK_I_ consistently exhibits a larger standard deviation compared to MK_A_.

	*Unit*	A	B	C	D
*Q*_3_	%	85	97	99	96
*Q*_6_	%	16	59	85	76
Δ*A*(L)	%	0.1 ± 2.3	–0.1 ± 1.6	0.1 ± 0.9	–0.2 ± 1.9
Δ*A*(S)	%	0.0 ± 1.9	–0.0 ± 1.4	0.0 ± 0.9	–0.2 ± 1.3
ΔMD	μm^2^/ms	0.00 ± 0.16	0.00 ± 0.10	0.00 ± 0.07	0.00 ± 0.12
ΔMK_I_		0.00 ± 0.64	0.02 ± 0.44	–0.02 ± 0.29	0.00 ± 0.39
ΔMK_A_		0.01 ± 0.37	0.00 ± 0.25	0.01 ± 0.16	–0.03 ± 0.25
ΔμFA		0.00 ± 0.07	0.00 ± 0.05	0.00 ± 0.03	0.00 ± 0.05

**Fig 4:**
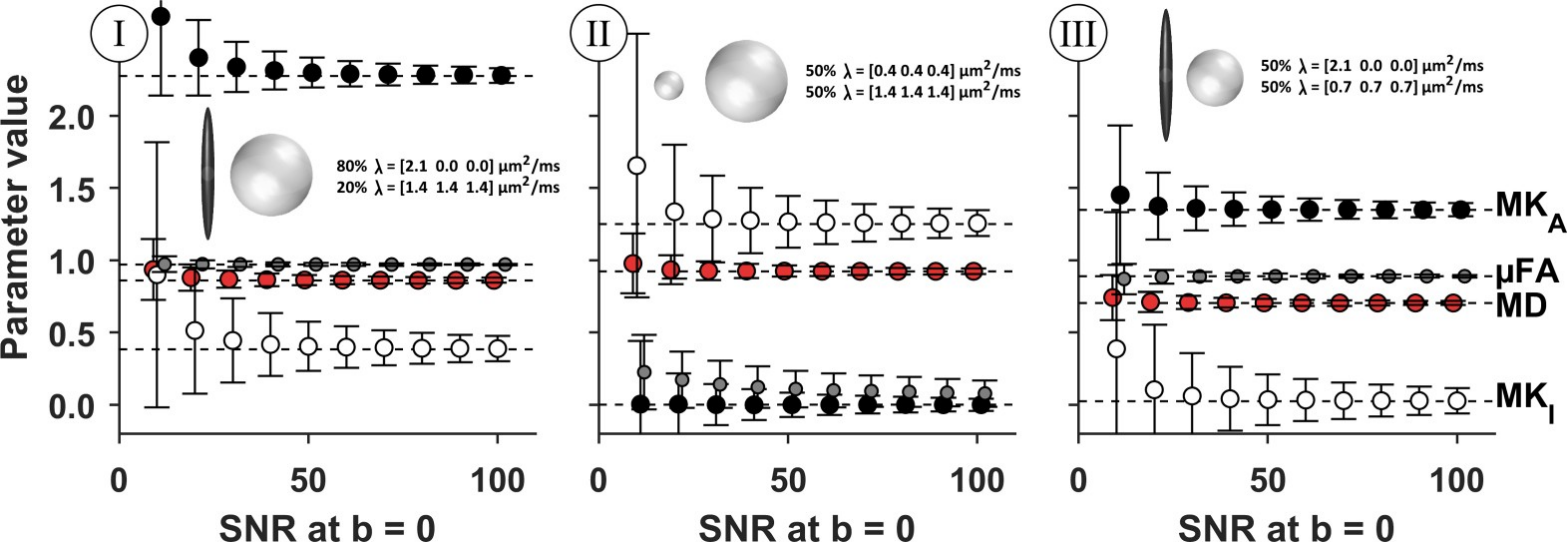
Simulation of DIVIDE parameter accuracy and precision in three model tissues. Markers show the mean parameter value, and the whiskers show one standard deviation across 10^4^ independent realizations of noise. The dashed horizontal lines show the parameter values that are estimated for a noise-free signal; deviation from the line indicates parameter bias caused by noise. As expected, precision and accuracy both improve with increasing SNR. The estimation of MD and μFA appear to be the most accurate and precise, although the μFA shows a deterioration of accuracy and precision when its true value is low (panel II) [19]. Both MK_A_ and MK_I_ suffer a positive bias when SNR in the b = 0 image is approximately 20 or less. Interestingly, MK_A_ is always more precise and accurate than MK_I_, indicating that it is generally less sensitive to noise. The simulations show that signal noise can cause negative values for both MK_I_ and MK_A_, which is especially likely when the true values are close to zero. Finally, in the case where MK_I_ = 0 (panel III), signal noise did not cause a strong positive bias in MK_I_ for SNR levels that match configuration C (where the majority of voxels have SNR > 20 at *b* = 0.1 ms/μm^2^, Fig 3). This suggests that signal noise alone is not likely to explain the positive MK_I_ that is observed throughout the brain parenchyma.

Fig 4 shows the parameters estimated from simulations of noisy signal. The results show an association between SNR and parameter accuracy as well as precision. Overall, signal noise is a plausible cause for the parameter variations seen in the parameter maps in vivo, including occurrences of negative diffusional variance, especially at low SNR and for true values close to zero. The precision in MK_I_ was consistently lower than for MK_A_, regardless of their true values, in accordance with in vivo results (Table 2). Moreover, the simulations suggested that regions where SNR is low may render a positive parameter bias, especially in MK_I_. None of the tested cases showed that noise can induce a negative bias in MK_I_. Simulations of tissue where the true MK_I_ = 0 indicated that noise is unlikely to account for the positive MK_I_ values found throughout the brain. This indicates that the true isotropic diffusional variance is detectable and positive in brain parenchyma.

**Fig 5:**
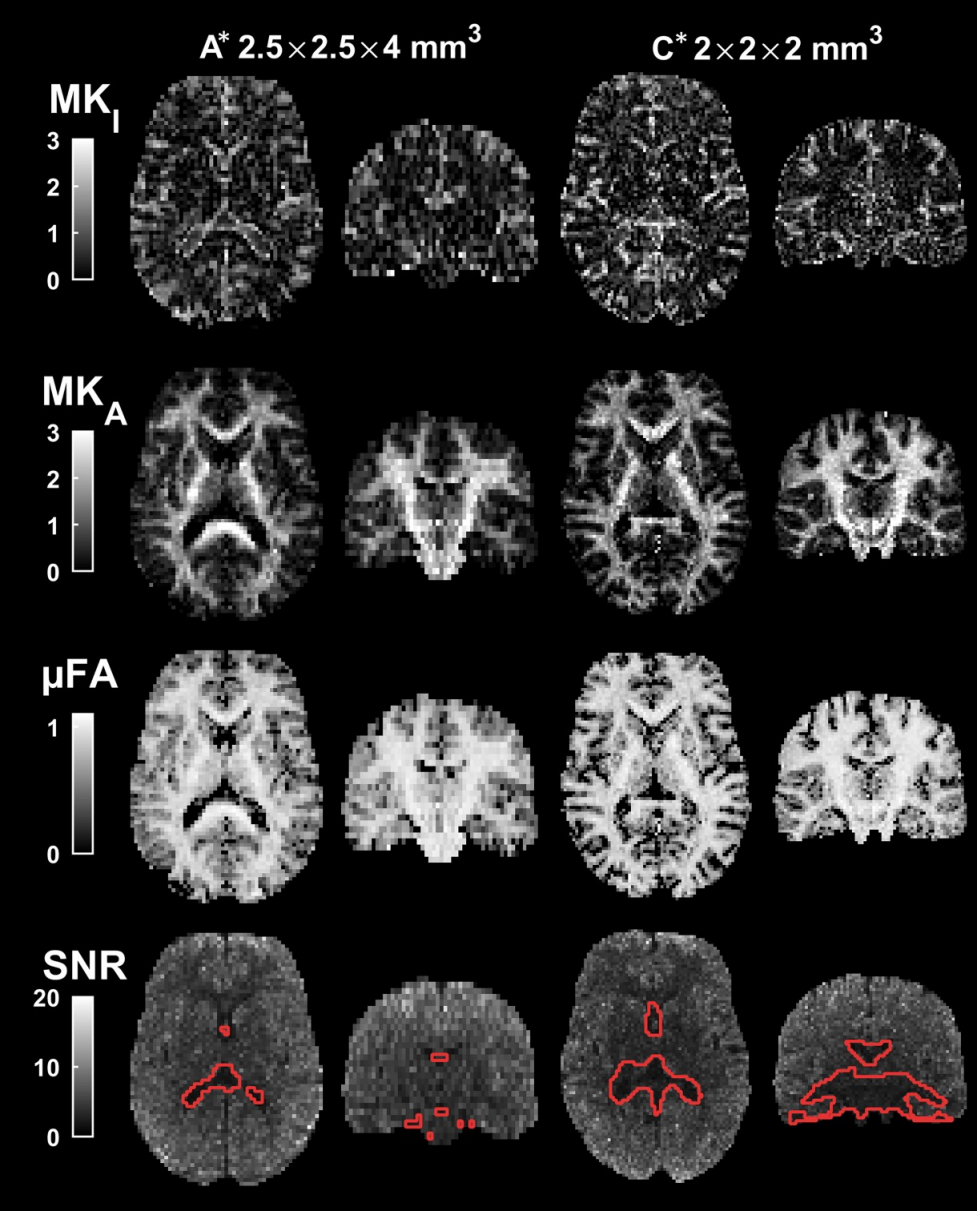
DIVIDE parameters and SNR maps for configurations A* and C*. The parameter maps from configuration A* are markedly less noisy than at the original resolution shown in Fig 2. For configuration C*, the resolution was increased, while maintaining high SNR in the superior and peripheral parts of the brain, although inferior and central parts showed regions where SNR was below 3 and may therefore suffer from non-negligible signal bias.

Resulting parameter and SNR maps at lower and higher resolutions on configurations A* and C*, are shown in Fig 5. As expected, the larger voxels used at configuration A* improved the SNR. For the lower resolution (2.5×2.5×4 mm^3^ voxels), we found *Q*_3_ = 99%, *Q*_6_ = 61%, compared to *Q*_3_ = 85%, *Q*_6_ = 16% with the baseline resolution (2×2×4 mm^3^ voxels). For configuration C*, the smaller voxels (2×2×2 mm^3^) reduced the overall SNR, but it remained relatively high at *Q*_3_ = 94% and *Q*_6_ = 45%. These results show that protocols with image resolutions tailored to the system performance are also technically feasible.

**Fig 6:**
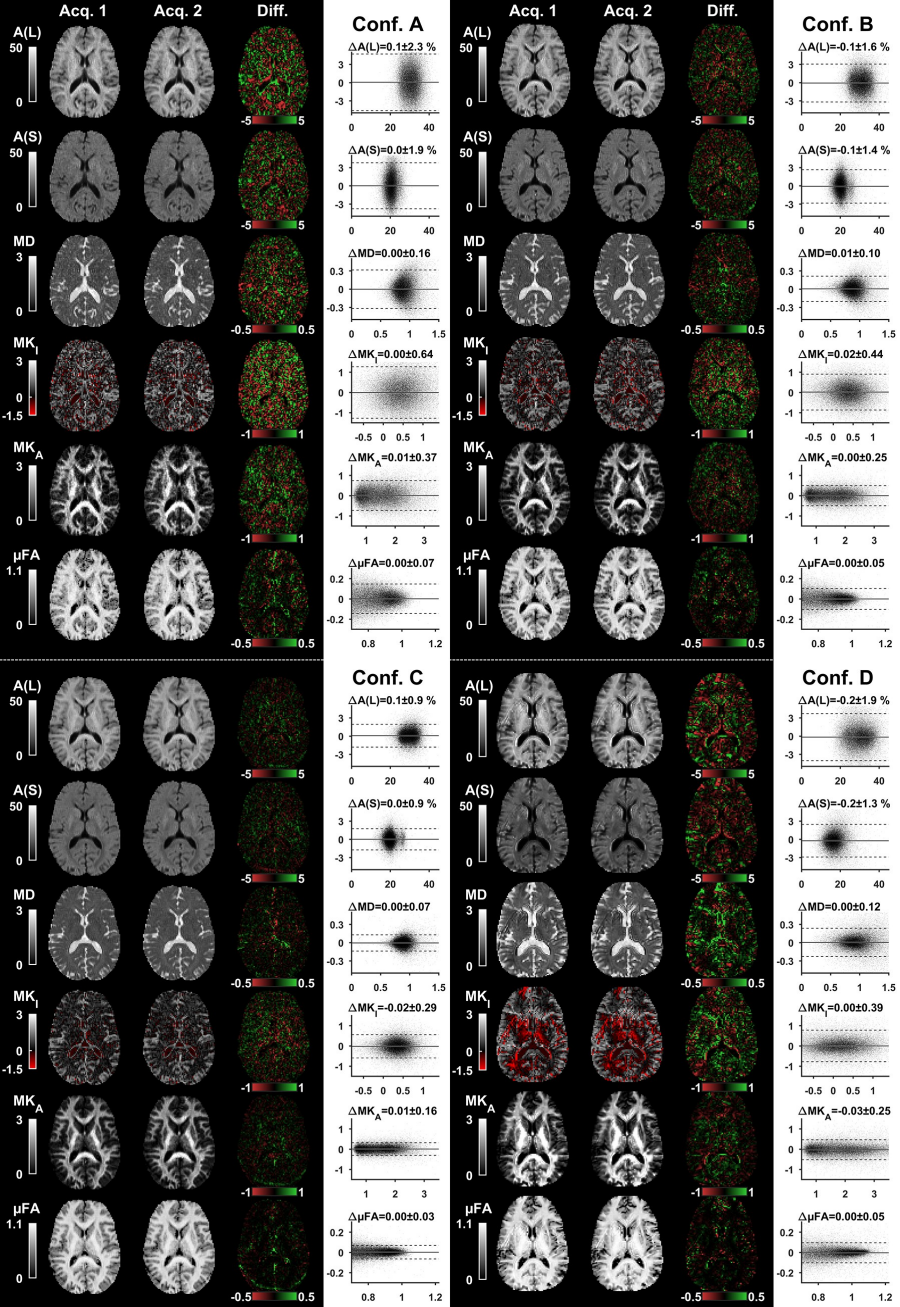
**Parameter maps from repeated acquisitions and analysis of repeatability for configurations A-D.** The voxel-wise difference (Diff.) between the first and second acquisition (Acq. 1 and 2) is color coded in red-green. The normalized powder-averaged signal at *b* = 2 ms/μm^2^ (*A*(L) and *A*(S)) is given in percent, and the MD is given in units of μm^2^/ms. The difference maps show the largest differences in tissue interfaces where small misregistration between the first and second acquisition causes large parameter discrepancy. Bland-Altman plots show the distributions of voxel-wise differences in tissue where μFA < 0.7 and MD < 1.5 μm^2^/ms. Solid and dashed lines show the average and two standard deviations of the distributions. All configurations showed negligible bias in repeatability of signal and DIVIDE parameters, and a configuration-dependent parameter precision. Note that the estimated precision pertains to the per-voxel parameter uncertainty; analyzing the average over multiple voxels is expected to markedly improve the precision.

Fig 6 shows the repeatability analysis for configurations A-D. The parameter maps for the first and second acquisition are qualitatively similar; the differences are shown in transversal slices and visualized in Bland-Altman plots. The highest repeatability was observed for configuration C for all included parameters. MK_I_ consistently exhibited the lowest precision, in agreement with the simulations (Fig 4). Notably, the precision of MD and μFA are both visibly dependent on their absolute values, which warrants a careful interpretation of the global standard deviation. The mean and standard deviation of voxel-wise differences are reported in Table 2.

## Discussion

We have demonstrated that tensor-valued diffusion encoding with high b-values is technically feasible across a wide range of different MRI scanners and configurations, and we have shown the range of results that can be expected in terms of DIVIDE parameter maps. These results can serve as a baseline in the design of future experiments. In addition to presenting imaging protocols with high efficiency, this work also demonstrates that scanners with lower gradient strengths—not commonly associated with ‘advanced diffusion imaging’—have sufficient performance and stability for tensor-valued diffusion encoding by asymmetric gradient waveforms at reasonable SNR and resolution. Even at 1.5 T and a maximum gradient waveform amplitude of 33 mT/m (configuration A), a minor reduction in spatial resolution was enough to facilitate relatively high SNR. Likewise, the high performance of configuration C could be harnessed to access a higher resolution, albeit at a prolonged scan time related to an increase in number of slices (Fig 5).

We acknowledge that the present implementation at configuration D (7 T) displays a prominent inconsistency, namely repeatable regions where MK_I_ is negative, which are not physical and cannot be explained by low SNR (Fig 4). The current results cannot be contrasted to literature since, to the best of our knowledge, b-tensor encoding at 7 T has not been investigated on a group level in vivo. We speculate that this may be caused by, subject motion or poor eddy-current compensation. It may also be related to field-dependent relaxation times in different tissue compartments [59–62] which may cause large values of MK_A_ and an underestimation of MK_I_, predicated on a longer decay time in the most anisotropic compartment. Although further investigations are warranted to understand the source and impact of this bias, data suggests that tensor-valued diffusion encoding is feasible also at ultra-high fields if the bias can be neglected or addressed.

Simulations based on realistic signal characteristics and SNR levels established a relation between SNR and the accuracy and precision of DIVIDE parameters. As shown in the parameter maps and simulations, low SNR introduces a positive parameter bias, most prominently seen for MK_I_. This is especially relevant in the central parts of the brain where the SNR tends to be lower compared to peripheral tissue. The simulations also showed that noise had a markedly smaller impact on MK_A_ compared with MK_I_, a feature that can be appreciated also in the parameter maps.

The technical feasibility, especially in a clinical setting, hinges mainly on the use of parsimonious sampling protocols and efficient gradient waveforms. In this study we achieved efficient tensor-valued encoding by using optimized asymmetric gradient waveforms [20]. The benefit of using the currently proposed waveforms and protocols can be appreciated by comparing them to our initial implementation [32], where a 10-minute protocol at a 3T scanner with 80 mT/m gradients covered only 5 slices at a resolution of 3×3×3 mm^3^. For reference, using the previous waveform design [18] at configurations A, C, and D would render echo times of approximately 230, 140 and 170 ms, respectively. This translates to a loss in SNR of 60%, 50% and 70%, assuming white matter relaxation times presented by Cox and Gowland [33]. Although asymmetric designs excel in efficiency, they are susceptible to errors caused by concomitant fields [63, 64]. Such errors can be difficult to detect by the naked eye, especially in vivo, but lead to quantification errors for several popular asymmetric gradient designs. To avoid this, we used so called Maxwell-compensated waveforms [40].

We emphasize that this study surveys technical feasibility, whereas investigation of the *clinical* feasibility, pertaining to specific applications, was outside the scope of this work. Preliminary work suggests that tensor-valued diffusion encoding may add novel information to investigations of schizophrenia [21], brain tumors [9], multiple sclerosis [28, 65], cortical malformations [66], prostate tumors [67], microstructure imaging of the healthy brain [32, 68], and kidneys [69]. The present protocol design was intended for investigations of healthy brain tissue, and therefore accounts for the approximate diffusivity and anisotropy of brain tissue, as described in the Supporting Information. Naturally, investigations of tissues with vastly different characteristics may require a different protocol. For example, a protocol designed for tumors where MD ≈ 1.8 μm^2^/ms and FA ≈ 0.1 [9] would generally entail a lower maximal b-value (*b*_max_ < 1.3 ms/μm^2^) and fewer diffusion-encoding directions (*n*_min_ = 3 at *b*_max_)—a markedly different premise for optimization compared to normal brain tissue (Supporting Information). A comprehensive protocol design should also consider the relaxation characteristics of the tissue. For example, transversal relaxation rates and SNR may depend on the tissue iron content [70, 71], which is associated with ageing. Specific applications can also benefit substantially from adapting the image quality and scan time to the expected level of variance in the studied population [72]. Tensor-valued diffusion encoding can be accelerated by simultaneous multi-slice imaging [73] and interleaving techniques [36] that reduce acquisition times and improve duty cycle constraints, as well as improvement in terms of reducing eddy-current effects [74, 75].

In this study we used DIVIDE to investigate repeatability of parameters related to tissue microstructure which have a straightforward interpretation if effects of diffusion time and intra-compartment kurtosis are both negligible. Although studies of the brain performed at relatively long diffusion times indicate that such effects are subtle [76–78], we emphasize that the multi-Gaussian framework may be incomplete [79], as demonstrated by specialized diffusion encoding schemes that have detected time-dependent diffusion coefficients in biological tissues [80–85]. Future studies will aim to design waveforms to probe the interplay between diffusion-time dependence, compartment kurtosis and exchange [41, 79, 86, 87].

We acknowledge several limitations of the present study. First, we used asymmetric voxels (2×2×4 mm^3^) as an effective means to increase the SNR and coverage of the protocols. Asymmetric voxels are known to introduce confounding effects in measures of voxel-scale diffusion anisotropy, for example in DTI, due to the interaction between voxel and structure geometry [88]. A similar limitation is not true for DIVIDE since it renders parameters that are independent of the orientation dispersion of the underlying tissue [19]. However, other issues pertaining to asymmetric voxels remain, such as increased partial-volume effects in the through-plane direction. This is especially relevant if the data is also intended to support tractography, where isotropic spatial resolution is preferable [89, 90]. Second, this study aimed to represent a wide range of MRI scanners, but several relevant hardware features were omitted in this study. For example, we did not account for the effects of coil design, RF and gradient systems, field heterogeneity, shimming, image acquisition, or reconstruction technique [34, 57, 58, 91]. Furthermore, the settings for the waveform optimization are not likely to be optimal because optimality depends on experimental settings, hardware configuration, and duty cycle, all of which may be site specific. Nevertheless, the waveforms suggested herein may serve as a starting point for future optimization. Finally, we emphasize that although the included MRI systems span a wide range of capabilities, current results are based on a single healthy volunteer, a single analysis method, post-processing pipeline, and a limited set of scanners, and may therefore lack generalizability. However, two critical aspects of technical feasibility and robustness [92], namely repeatability and sufficiently high SNR, could be established. The test-retest data in Fig 6 show that the tensor-valued encoding yields repeatable results for all configurations, except for the normalized signal for configuration D. The generalizability can also be strengthened by a separate analysis that we performed on a cohort of ten subjects, previously acquired with hardware specifications similar to configuration B and used in a proof-of-concept implementation [20]. Although that study had limited coverage (44 mm feet-head) and longer echo time (TE = 130 ms), the experiments yielded homogeneous data quality on the group level, where *Q*_3_ = 95 ± 2% and *Q*_6_ = 21 ± 4%; consistent with the current quality estimated at configuration B when considering the longer TE.

## Conclusions

Tensor-valued diffusion encoding can probe microstructural features beyond those available with conventional methods, but it requires non-conventional diffusion-encoding waveforms. The preferred platform for in vivo experiments based on such experiments has been 3 T scanners with high-performance gradients. In this study, we demonstrated that tensor-valued encoding that supports DIVIDE analysis is technically feasible over a wide range of MRI scanners, with main magnetic fields between 1.5 and 7 T, and gradient waveform amplitudes as low as 33 mT/m. We have also reported baseline repeatability values for whole-brain DIVIDE protocols with acquisition times between 5 and 9 minutes. The implementation was facilitated by efficient asymmetric gradient waveforms and parsimonious signal sampling protocols. By enabling tensor-valued diffusion encoding and DIVIDE at a wide range of scanners at clinically acceptable acquisition times, we expect that such methods may be more broadly used to facilitate new and exciting venues for dMRI research.

## Supporting information

S1 Fig Minimal number of directions (*n*_min_) required to yield a rotation invariant powder-averaged signal for linear and planar tensor encoding (LTE and PTE). *n*_min_ can be read from the figure for combinations of the tissue fractional anisotropy (FA) and attenuation factor (*b*·MD), where the numbers in the circles show *n*_min_ for a color-coded interval. As expected, higher anisotropy and attenuation both demand a larger number of diffusion-encoding directions. Spherical tensor encoding always requires *n*_min_ = 1, since it is inherently invariant to rotation. Note that we consider the signal to be rotation invariant when CV < 1%. The PTE plot is included for completeness, even if it was not used in the data acquisition of this study.
(DOCX)

## References

[1] Stejskal EO. Use of Spin Echoes in a Pulsed Magnetic-Field Gradient to Study Anisotropic, Restricted Diffusion and Flow. The Journal of Chemical Physics. 1965;43(10):3597 10.1063/1.1696526

[2] Basser PJ, Mattiello J, Le Bihan D. MR diffusion tensor spectroscopy and imaging. Biophys J. 1994;66(1):259–67. 10.1016/S0006-3495(94)80775-1 8130344 PMC1275686

[3] Assaf Y, Pasternak O. Diffusion tensor imaging (DTI)-based white matter mapping in brain research: a review. J Mol Neurosci. 2008;34(1):51–61. 10.1007/s12031-007-0029-0 . 18157658

[4] Jensen JH, Helpern JA, Ramani A, Lu H, Kaczynski K. Diffusional kurtosis imaging: the quantification of non-gaussian water diffusion by means of magnetic resonance imaging. Magn Reson Med. 2005;53(6):1432–40. 10.1002/mrm.20508 . 15906300

[5] Raab P, Hattingen E, Franz K, Zanella FE, Lanfermann H. Cerebral gliomas: diffusional kurtosis imaging analysis of microstructural differences. Radiology. 2010;254(3):876–81. 10.1148/radiol.09090819 . 20089718

[6] Van Cauter S, Veraart J, Sijbers J, Peeters RR, Himmelreich U, De Keyzer F, et al Gliomas: diffusion kurtosis MR imaging in grading. Radiology. 2012;263(2):492–501. 10.1148/radiol.12110927 . 22403168

[7] Novikov DS, Kiselev VG, Jespersen SN. On modeling. Magn Reson Med. 2018 Epub 2018/03/02. 10.1002/mrm.27101 . 29493816 PMC5905348

[8] Mitra P. Multiple wave-vector extensions of the NMR pulsed-field-gradient spin-echo diffusion measurement. Physical Review B. 1995;51(21):15074–8. 10.1103/PhysRevB.51.15074 9978461

[9] Szczepankiewicz F, van Westen D, Englund E, Westin CF, Stahlberg F, Latt J, et al The link between diffusion MRI and tumor heterogeneity: Mapping cell eccentricity and density by diffusional variance decomposition (DIVIDE). Neuroimage. 2016;142:522–32. 10.1016/j.neuroimage.2016.07.038 27450666 PMC5159287

[10] Jespersen SN. Equivalence of double and single wave vector diffusion contrast at low diffusion weighting. NMR Biomed. 2012;25(6):813–8. Epub 2011/12/03. 10.1002/nbm.1808 . 22134913

[11] Henriques RN, Jespersen SN, Shemesh N. Microscopic anisotropy misestimation in spherical-mean single diffusion encoding MRI. Magn Reson Med. 2019 Epub 2019/01/17. 10.1002/mrm.27606 . 30648753 PMC6519215

[12] Cory DG, Garroway AN, Miller JB. Applications of Spin Transport as a Probe of Local Geometry. Abstr Pap Am Chem S. 1990;199:105. WOS:A1990CZ78501175.

[13] Shemesh N, Özarslan E, Komlosh ME, Basser PJ, Cohen Y. From single-pulsed field gradient to double-pulsed field gradient MR: gleaning new microstructural information and developing new forms of contrast in MRI. NMR Biomed. 2010;23(7):757–80. 10.1002/nbm.1550 20690130 PMC3139994

[14] Mori S, van Zijl P. Diffusion Weighting by the Trace of the Diffusion Tensor within a Single Scan. Magn Reson Med. 1995;33(1):41–52. 10.1002/mrm.1910330107 WOS:A1995PZ80800006. 7891534

[15] Wong EC, Cox RW, Song AW. Optimized isotropic diffusion weighting. Magn Reson Med. 1995;34(2):139–43. . 7476070 10.1002/mrm.1910340202

[16] Butts K, Pauly J, de Crespigny A, Moseley M. Isotropic diffusion-weighted and spiral-navigated interleaved EPI for routine imaging of acute stroke. Magn Reson Med. 1997;38(5):741–9. Epub 1997/11/14. . 9358448 10.1002/mrm.1910380510

[17] Ianus A, Shemesh N. Incomplete initial nutation diffusion imaging: An ultrafast, single-scan approach for diffusion mapping. Magn Reson Med. 2017 Epub 2017/09/05. 10.1002/mrm.26894 . 28868785 PMC5836954

[18] Eriksson S, Lasič S, Topgaard D. Isotropic diffusion weighting in PGSE NMR by magic-angle spinning of the q-vector. J Magn Reson. 2013;226:13–8. 10.1016/j.jmr.2012.10.015 . 23178533

[19] Lasič S, Szczepankiewicz F, Eriksson S, Nilsson M, Topgaard D. Microanisotropy imaging: quantification of microscopic diffusion anisotropy and orientational order parameter by diffusion MRI with magic-angle spinning of the q-vector. Frontiers in Physics. 2014;2:11 10.3389/fphy.2014.00011

[20] Sjölund J, Szczepankiewicz F, Nilsson M, Topgaard D, Westin CF, Knutsson H. Constrained optimization of gradient waveforms for generalized diffusion encoding. J Magn Reson. 2015;261:157–68. 10.1016/j.jmr.2015.10.012 . 26583528 PMC4752208

[21] Westin CF, Knutsson H, Pasternak O, Szczepankiewicz F, Özarslan E, van Westen D, et al Q-space trajectory imaging for multidimensional diffusion MRI of the human brain. Neuroimage. 2016;135:345–62. 10.1016/j.neuroimage.2016.02.039 26923372 PMC4916005

[22] Topgaard D. Multidimensional diffusion MRI. J Magn Reson. 2017;275:98–113. 10.1016/j.jmr.2016.12.007 . 28040623

[23] Özarslan E, Basser PJ. Microscopic anisotropy revealed by NMR double pulsed field gradient experiments with arbitrary timing parameters. J Chem Phys. 2008;128(15):154511 10.1063/1.2905765 18433239 PMC2809669

[24] Jespersen SN, Lundell H, Sønderby CK, Dyrby TB. Orientationally invariant metrics of apparent compartment eccentricity from double pulsed field gradient diffusion experiments. NMR Biomed. 2013;26(12):1647–62. 10.1002/nbm.2999 . 24038641

[25] Lawrenz M, Finsterbusch J. Double-wave-vector diffusion-weighted imaging reveals microscopic diffusion anisotropy in the living human brain. Magn Reson Med. 2013;69(4):1072–82. 10.1002/mrm.24347 . 22711603

[26] Avram AV, Ozarslan E, Sarlls JE, Basser PJ. In vivo detection of microscopic anisotropy using quadruple pulsed-field gradient (qPFG) diffusion MRI on a clinical scanner. Neuroimage. 2013;64:229–39. 10.1016/j.neuroimage.2012.08.048 22939872 PMC3520437

[27] Ianus A, Drobnjak I, Alexander DC. Model-based estimation of microscopic anisotropy using diffusion MRI: a simulation study. NMR Biomed. 2016;29(5):672–85. 10.1002/nbm.3496 . 27003223

[28] Yang G, Tian Q, Leuze C, Wintermark M, McNab JA. Double diffusion encoding MRI for the clinic. Magn Reson Med. 2017 Epub 2017/12/22. 10.1002/mrm.27043 . 29266375 PMC5910247

[29] Jensen JH, Hui ES, Helpern JA. Double-pulsed diffusional kurtosis imaging. NMR Biomed. 2014;27:363–70. 10.1002/nbm.3030 . 24623712

[30] Shemesh N, Ozarslan E, Adiri T, Basser PJ, Cohen Y. Noninvasive bipolar double-pulsed-field-gradient NMR reveals signatures for pore size and shape in polydisperse, randomly oriented, inhomogeneous porous media. J Chem Phys. 2010;133(4):044705 10.1063/1.3454131 20687674 PMC2921441

[31] Lawrenz M, Koch MA, Finsterbusch J. A tensor model and measures of microscopic anisotropy for double-wave-vector diffusion-weighting experiments with long mixing times. J Magn Reson. 2010;202(1):43–56. 10.1016/j.jmr.2009.09.015 . 19854085

[32] Szczepankiewicz F, Lasič S, van Westen D, Sundgren PC, Englund E, Westin CF, et al Quantification of microscopic diffusion anisotropy disentangles effects of orientation dispersion from microstructure: Applications in healthy volunteers and in brain tumors. Neuroimage. 2015;104:241–52. 10.1016/j.neuroimage.2014.09.057 . 25284306 PMC4252798

[33] Cox EF, Gowland PA. Simultaneous quantification of T2 and T'2 using a combined gradient echo-spin echo sequence at ultrahigh field. Magn Reson Med. 2010;64(5):1440–5. 10.1002/mrm.22522 . 20593370

[34] Choi S, Cunningham DT, Aguila F, Corrigan JD, Bogner J, Mysiw WJ, et al DTI at 7 and 3 T: systematic comparison of SNR and its influence on quantitative metrics. Magn Reson Imaging. 2011;29(6):739–51. 10.1016/j.mri.2011.02.009 . 21571473

[35] Szczepankiewicz F, Westin CF, Ståhlberg F, Lätt J, Nilsson M, editors. Microscopic Anisotropy Imaging at 7T Using Asymmetrical Gradient Waveform Encoding. Proc Intl Soc Mag Reson Med 24; 2016; Singapore.

[36] Hutter J, Nilsson M, Christiaens D, Schneider T, Price AN, Hajnal JV, et al, editors. Highly efficient diffusion MRI by slice-interleaved free-waveform imaging (SIFI). ISMRM; 2018; Paris, France.

[37] Vos SB, Tax CM, Luijten PR, Ourselin S, Leemans A, Froeling M. The importance of correcting for signal drift in diffusion MRI. Magn Reson Med. 2017;77(1):285–99. Epub 2016/01/30. 10.1002/mrm.26124 . 26822700

[38] Ham CL, Engels JM, van de Wiel GT, Machielsen A. Peripheral nerve stimulation during MRI: effects of high gradient amplitudes and switching rates. J Magn Reson Imaging. 1997;7(5):933–7. . 9307922 10.1002/jmri.1880070524

[39] Hebrank FX, Gebhardt M, editors. SAFE-Model—A New Method for Predicting Peripheral Nerve Stimulations in MRI. Proc Intl Soc Mag Res Med; 2000.

[40] Szczepankiewicz F, Nilsson M, editors. Maxwell-compensated waveform design for asymmetric diffusion encoding. Proc Intl Soc Mag Reson Med 26; 2018; Paris, France. 10.1002/mrm.27828 PMC6626569 31148245

[41] Lundell H, Nilsson M, Dyrby TB, Parker GJ, Hubbard P, Zhou F, et al Microscopic anisotropy with spectrally modulated q-space trajectory encoding. Proc Intl Soc Mag Reson Med 25; Honolulu, Hawaii2017.

[42] Klein S, Staring M, Murphy K, Viergever MA, Pluim JP. elastix: a toolbox for intensity-based medical image registration. IEEE Trans Med Imaging. 2010;29(1):196–205. 10.1109/TMI.2009.2035616 . 19923044

[43] Nilsson M, Szczepankiewicz F, van Westen D, Hansson O. Extrapolation-Based References Improve Motion and Eddy-Current Correction of High B-Value DWI Data: Application in Parkinson's Disease Dementia. PLoS ONE. 2015;10(10):e0141825 10.1371/journal.pone.0141825 . 26528541 PMC4631453

[44] Yablonskiy DA, Sukstanskii AL. Theoretical models of the diffusion weighted MR signal. NMR Biomed. 2010;23(7):661–81. 10.1002/nbm.1520 . 20886562 PMC6429954

[45] Nilsson M, Englund E, Szczepankiewicz F, van Westen D, Sundgren PC. Imaging brain tumour microstructure. Neuroimage. 2018 Epub 2018/05/12. 10.1016/j.neuroimage.2018.04.075 . 29751058

[46] Jensen JH, Helpern JA. MRI quantification of non-Gaussian water diffusion by kurtosis analysis. NMR Biomed. 2010;23(7):698–710. 10.1002/nbm.1518 20632416 PMC2997680

[47] Röding M, Bernin D, Jonasson J, Sarkka A, Topgaard D, Rudemo M, et al The gamma distribution model for pulsed-field gradient NMR studies of molecular-weight distributions of polymers. J Magn Reson. 2012;222:105–11. 10.1016/j.jmr.2012.07.005 . 22864268

[48] Topgaard D. NMR methods for studying microscopic diffusion anisotropy In: Valiullin R, editor. Diffusion NMR in Confined Systems: Fluid Transport in Porous Solids and Heterogeneous Materials. New Developments in NMR: Royal Society of Chemistry, Cambridge, UK; 2016.

[49] Szczepankiewicz F, Sjölund J, Ståhlberg F, Lätt J, Nilsson M. Whole-brain diffusional variance decomposition (DIVIDE): Demonstration of technical feasibility at clinical MRI systems. arXiv:161206741. 2016. 10.1371/journal.pone.0214238 PMC6438503 30921381

[50] Lawrenz M, Brassen S, Finsterbusch J. Microscopic diffusion anisotropy in the human brain: reproducibility, normal values, and comparison with the fractional anisotropy. Neuroimage. 2015;109:283–97. 10.1016/j.neuroimage.2015.01.025 . 25595503

[51] Nilsson M, Szczepankiewicz F, Lampinen B, Ahlgren A, De Almeida Martins JP, Lasic S, et al, editors. An open-source framework for analysis of multidimensional diffusion MRI data implemented in MATLAB. Proc Intl Soc Mag Reson Med 26; 2018; Paris, France.

[52] Bartlett JW, Frost C. Reliability, repeatability and reproducibility: analysis of measurement errors in continuous variables. Ultrasound Obstet Gynecol. 2008;31(4):466–75. 10.1002/uog.5256 . 18306169

[53] Jones DK, Basser PJ. "Squashing peanuts and smashing pumpkins": how noise distorts diffusion-weighted MR data. Magn Reson Med. 2004;52(5):979–93. 10.1002/mrm.20283 . 15508154

[54] Dietrich O, Raya JG, Reeder SB, Ingrisch M, Reiser MF, Schoenberg SO. Influence of multichannel combination, parallel imaging and other reconstruction techniques on MRI noise characteristics. Magn Reson Imaging. 2008;26(6):754–62. 10.1016/j.mri.2008.02.001 . 18440746

[55] Sjölund J, Eklund A, Özarslan E, Herberthson M, Bankestad M, Knutsson H. Bayesian uncertainty quantification in linear models for diffusion MRI. Neuroimage. 2018;175:272–85. Epub 2018/04/01. 10.1016/j.neuroimage.2018.03.059 . 29604453 PMC6419970

[56] Gudbjartsson H, Patz S. The Rician Distribution of Noisy MRI Data. Magn Reson Imaging. 1995;34(6):910–4. 10.1002/mrm.1910340618 PMC2254141 8598820

[57] Sigmund EE, Gutman D. Diffusion-weighted imaging of the brain at 7 T with echo-planar and turbo spin echo sequences: preliminary results. Magn Reson Imaging. 2011;29(6):752–65. 10.1016/j.mri.2011.02.016 . 21550741

[58] Moser E, Ståhlberg F, Ladd ME, Trattnig S. 7-T MR—from research to clinical applications? NMR Biomed. 2012;25(5):695–716. 10.1002/nbm.1794 . 22102481

[59] Veraart J, Fieremans E, Novikov DS, editors. Quantifying neuronal microstructure integrity with TE dependent Diffusion Imaging (TEdDI). Proc Intl Soc Mag Reson Med 25; 2017; Honolulu, USA.

[60] Qin W, Yu CS, Zhang F, Du XY, Jiang H, Yan YX, et al Effects of echo time on diffusion quantification of brain white matter at 1.5 T and 3.0 T. Magn Reson Med. 2009;61(4):755–60. 10.1002/mrm.21920 . 19191286

[61] Tax CM, Rudrapatna U, Witzel T, Jones DK, editors. Disentangling in two dimensions in the living human brain: Feasbilty of relaxometry-diffusometry using ultra-strong gradients. Proc Intl Soc Mag Reson Med 25 2017; Honolulu, USA.

[62] Qiuyun F, Huang SY, Nummenmaa A, Witzel T, Wald L, editors. T2 relaxation rates of the fast and slow bi-exponential diffusion components in the in vivo corpus callosum. Proc Intl Soc Mag Reson Med 25; 2017; Honolulu, USA.

[63] Bernstein MA, Zhou XJ, Polzin JA, King KF, Ganin A, Pelc NJ, et al Concomitant gradient terms in phase contrast MR: analysis and correction. Magn Reson Med. 1998;39(2):300–8. . 9469714 10.1002/mrm.1910390218

[64] Baron CA, Lebel RM, Wilman AH, Beaulieu C. The effect of concomitant gradient fields on diffusion tensor imaging. Magn Reson Med. 2012;68(4):1190–201. 10.1002/mrm.24120 . 22851517

[65] Winther Andersen K, Lasic S, Lundell H, Nilsson M, Topgaard D, Szczepankiewicz F, et al, editors. Multi-dimensional microstructural imaging offers novel in vivo insights into brain pathology: an application to multiple sclerosis. Proc Intl Soc Mag Reson Med 26; 2018; Paris, France.

[66] Lampinen B, Zampeli A, Szczepankiewicz F, Compagno Strandberg M, Källen K, Björkman-Burtschen IM, et al, editors. Microscopic diffusion anisotorpy reveals microstructural heterogeneity of malformations of cortical development associated with epilepsy: A b-tensor encoding study at 7 T. Proc Intl Soc Mag Reson Med 26; 2018; Paris, France.

[67] Nilsson M, Szczepankiewicz F, Skorpil M, Westin CF, Blomqvist L, Jäderling F, editors. Mapping prostatic microscopic anisotropy using linear and spherical b-tensor encoding: A preliminary study. Proc Intl Soc Mag Reson Med 25; 2017; Singapore. 10.1002/mrm.28856 PMC9272946 34056750

[68] Lampinen B, Szczepankiewicz F, Martensson J, van Westen D, Sundgren PC, Nilsson M. Neurite density imaging versus imaging of microscopic anisotropy in diffusion MRI: A model comparison using spherical tensor encoding. Neuroimage. 2017;147:517–31. 10.1016/j.neuroimage.2016.11.053 . 27903438

[69] Nery F, Hall MG, Thomas DL, Kaden E, Szczepankiewicz F, Gordon I, et al, editors. Microscopic diffusion anisotropy imaging in the kidneys. Proc Intl Soc Mag Reson Med 26; 2018; Paris, France.

[70] Siemonsen S, Finsterbusch J, Matschke J, Lorenzen A, Ding XQ, Fiehler J. Age-dependent normal values of T2* and T2' in brain parenchyma. AJNR Am J Neuroradiol. 2008;29(5):950–5. 10.3174/ajnr.A0951 . 18272561 PMC8128565

[71] Gelman N, Gorell JM, Barker PB, Savage RM, Spickler EM, Windham JP, et al MR imaging of human brain at 3.0 T: preliminary report on transverse relaxation rates and relation to estimated iron content. Radiology. 1999;210(3):759–67. 10.1148/radiology.210.3.r99fe41759 . 10207479

[72] Szczepankiewicz F, Lätt J, Wirestam R, Leemans A, Sundgren P, van Westen D, et al Variability in diffusion kurtosis imaging: impact on study design, statistical power and interpretation. Neuroimage. 2013;76:145–54. 10.1016/j.neuroimage.2013.02.078 . 23507377

[73] Setsompop K, Cohen-Adad J, Gagoski BA, Raij T, Yendiki A, Keil B, et al Improving diffusion MRI using simultaneous multi-slice echo planar imaging. Neuroimage. 2012;63(1):569–80. 10.1016/j.neuroimage.2012.06.033 22732564 PMC3429710

[74] Aliotta E, Moulin K, Ennis DB. Eddy current-nulled convex optimized diffusion encoding (EN-CODE) for distortion-free diffusion tensor imaging with short echo times. Magn Reson Med. 2018;79(2):663–72. Epub 2017/04/27. 10.1002/mrm.26709 . 28444802

[75] Yang G, McNab JA. Eddy current nulled constrained optimization of isotropic diffusion encoding gradient waveforms. Magn Reson Med. 2018 Epub 2018/10/29. 10.1002/mrm.27539 . 30368913 PMC6347544

[76] Clark CA, Hedehus M, Moseley ME. Diffusion time dependence of the apparent diffusion tensor in healthy human brain and white matter disease. Magn Reson Med. 2001;45(6):1126–9. . 11378893 10.1002/mrm.1149

[77] Ronen I, Moeller S, Ugurbil K, Kim DS. Analysis of the distribution of diffusion coefficients in cat brain at 9.4 T using the inverse Laplace transformation. Magn Reson Imaging. 2006;24(1):61–8. 10.1016/j.mri.2005.10.023 . 16410179

[78] Nilsson M, Lätt J, Nordh E, Wirestam R, Ståhlberg F, Brockstedt S. On the effects of a varied diffusion time in vivo: is the diffusion in white matter restricted? Magn Reson Imaging. 2009;27(2):176–87. 10.1016/j.mri.2008.06.003 18657924

[79] Nørhøj Jespersen S, Lynge Olesen J, Ianuş A, Shemesh N. Effects of nongaussian diffusion on “isotropic diffusion”measurements: an ex-vivo microimaging and simulation study. J Magn Reson. 2019 10.1016/j.jmr.2019.01.007 30711786

[80] Stanisz GJ, Szafer A, Wright GA, Henkelman RM. An analytical model of restricted diffusion in bovine optic nerve. Magn Reson Med. 1997;37(1):103–11. . 8978638 10.1002/mrm.1910370115

[81] Assaf Y, Mayk A, Cohen Y. Displacement imaging of spinal cord using q-space diffusion-weighted MRI. Magn Reson Med. 2000;44(5):713–22. 10.1002/1522-2594(200011)44:5<713::Aid-Mrm9>3.0.Co;2-6 WOS:000165163600009. 11064406

[82] Does MD, Parsons EC, Gore JC. Oscillating gradient measurements of water diffusion in normal and globally ischemic rat brain. Magn Reson Med. 2003;49(2):206–15. 10.1002/mrm.10385 . 12541239

[83] Fieremans E, Burcaw LM, Lee HH, Lemberskiy G, Veraart J, Novikov DS. In vivo observation and biophysical interpretation of time-dependent diffusion in human white matter. Neuroimage. 2016 10.1016/j.neuroimage.2016.01.018 . 26804782 PMC4803645

[84] Lätt J, Nilsson M, van Westen D, Wirestam R, Ståhlberg F, Brockstedt S. Diffusion-weighted MRI measurements on stroke patients reveal water-exchange mechanisms in sub-acute ischaemic lesions. NMR Biomed. 2009;22(6):619–28. 10.1002/nbm.1376 . 19306340

[85] Grussu F, Ianus A, Tur C, Prados F, Schneider T, Kaden E, et al Relevance of time-dependence for clinically viable diffusion imaging of the spinal cord. Magn Reson Med. 2019;81(2):1247–64. Epub 2018/09/20. 10.1002/mrm.27463 . 30229564 PMC6586052

[86] Lundell H, Nilsson M, Westin CF, Topgaard D, Lasic S, editors. Spectral anisotropy in multidimensional diffusion encoding. Proc Intl Soc Mag Reson Med 26; 2018 3 20; Paris, France.

[87] Ning L, Setsompop K, Westin CF, Rathi Y. New Insights About Time-Varying Diffusivity and Its Estimation from Diffusion MRI. Magn Reson Med. 2016 10.1002/mrm.26403 . 27611013 PMC5344793

[88] Oouchi H, Yamada K, Sakai K, Kizu O, Kubota T, Ito H, et al Diffusion anisotropy measurement of brain white matter is affected by voxel size: underestimation occurs in areas with crossing fibers. AJNR Am J Neuroradiol. 2007;28(6):1102–6. 10.3174/ajnr.A0488 . 17569968 PMC8134156

[89] Jeurissen B, Szczepankiewicz F, editors. Spherical deconvolution of diffusion weighted data with tensor-valued encodings. Proc Intl Soc Mag Reson Med 26; 2018; Paris, France.

[90] Basser PJ, Pajevic S, Pierpaoli C, Duda J, Aldroubi A. In Vivo Fiber Tractography Using DT-MRI Data. Magn Reson Med. 2000;(44):625–32. 11025519 10.1002/1522-2594(200010)44:4<625::aid-mrm17>3.0.co;2-o

[91] Polders DL, Leemans A, Hendrikse J, Donahue MJ, Luijten PR, Hoogduin JM. Signal to noise ratio and uncertainty in diffusion tensor imaging at 1.5, 3.0, and 7.0 Tesla. J Magn Reson Imaging. 2011;33(6):1456–63. 10.1002/jmri.22554 . 21591016

[92] Andersson P. Robustness of Technical Systems in Relation to Quality, Reliability and Associated Concepts. Journal of Engineering Design. 1997;8(3):277–88. 10.1080/09544829708907966

