# Supplementary material for: Tensor-valued diffusion encoding for diffusional variance decomposition (DIVIDE): Technical feasibility in clinical MRI systems
Source: PLoS One. 2019 Mar 28;14(3):e0214238. doi: 10.1371/journal.pone.0214238 (PMC6438503; doi:10.1371/journal.pone.0214238)
Supplement: S1 Fig — nmin can be read from the figure for combinations of the tissue fractional anisotropy (FA) and attenuation factor (b·MD), where the numbers in the circles show nmin for a color-coded interval. As expected, higher anisotropy and attenuation both demand a larger number of diffusion-encoding directions. Spherical tensor encoding always requires nmin = 1, since it is inherently invariant to rotation. Note that we consider the signal to be rotation invariant when CV < 1%. The PTE plot is included for completeness, even if it was not used in the data acquisition of this study. (DOCX) [file pone.0214238.s001.docx]

# Supplementary information – Protocol design

The protocol design followed three steps. Step (i), set the minimal and maximal b-values (*b*_min_ and *b*_max_). The optimal value of *b*_max_ depends on several factors, but in the context of DKI, it has been suggested that a reasonable value is *b*_max_ = 2 ms/µm^2^ [1, 2], which was adopted in this study. An upper bound of the maximal b-value (*b*_max_) can also be established from the requirement that the phase dispersion is approximately Gaussian, which is fulfilled if the signal is not **attenuated** below 10%, that is if *S*(*b*_max_)/*S*_0_ > 0.1 [3]. For STE we observe maximal signal attenuation if MK_I_ = 0 (signal decay is mono-exponential), in which case

$b_{\max}<\frac{\ln\left( 10 \right)}{\mathrm{MD}},$ Eq. S1

gives the upper bound *b*_max_ < 2.3 ms/µm^2^ for MD ≈ 1 µm^2^/ms. The minimal b-value (*b*_min_) is commonly set to zero, however, to reduce the influence of incoherent intra-voxel motion [4], we set *b*_min_ = 0.1 ms/µm^2^.

Step (ii), set the TE, TR, and the number of samples. The TE was minimized under the constraint that the necessary *b*_max_ was attainable for all b-tensor shapes, and that the duty cycle was not violated. In this study we used a combination of LTE and STE because this combination renders the shortest TE and yields the largest signal contrast in the presence of microscopic diffusion anisotropy [5]. We note that STE is an efficient choice because it does not have to be rotated in order to yield rotation invariant signal, which can be exploited in optimization to yield a relatively high encoding efficiency [5], although other combinations may be beneficial if the duty cycle is the primary limitation. Several waveform designs for STE exist [6-8]. Here, we employed the optimized waveforms proposed by Sjölund et al. [9] due to their superior efficiency [5] and ability to compensate for concomitant gradients [10]. The TR was minimized based on the TE, number of slices, and the limitations imposed by the duty cycle. In principle, both TE and TR could be set to lower values, but using minimal values violated the duty cycle [9].

Step (iii), based on the configuration with the longest TR (configuration D), the number of measurements afforded by the total scan time were distributed over diffusion encoding directions, b-values and tensor shapes. We consider three constraints: a minimum of three unique b-values are required to invert the model in Eq. 1 in the main paper [1]; b-tensors with at least two unique $b_{\Delta}^{2}$ must be used to decompose the variance into its components [11]; and rotation invariance is achieved by sampling a sufficient number of well-distributed diffusion-encoding directions for each b-value [12], as described below. These three constraints, and an acquisition time of less than approximately 10 minutes, afforded the sampling protocol to include four b-values and 6, 6, 12 and 16 directions (or repetitions for STE). The model fit requires only three b-values but using four b-values is motivated by a higher robustness to situations where the signal accuracy at the highest b-values is impacted by the noise floor.

For each b-value, the minimum number of isotopically distributed diffusion-encoding directions required to yield a rotation invariant powder-average signal was estimated from simulations, according to the preliminary report by Szczepankiewicz et al. [13]. Briefly, we simulated the effects of rotation in a worst-case scenario, i.e., when using LTE in a model tissue described by a single diffusion tensor (**D**). The powder averaged signal ($\bar{S}$) was calculated as the average diffusion-weighted signal along a number (*n*_dir_) of diffusion encoding directions (**n**, |**n**| = 1), according to

$\bar{S}\left( b \right)=\frac{1}{n_{\mathrm{dir}}}\sum_{i=1}^{n_{\mathrm{dir}}} S_{0}\exp\left( -b\cdot\mathbf{n}_{i}\mathbf{D}\mathbf{n}_{i}^{T} \right),$ Eq. S2

where $\mathbf{n}_{i}$ is the *i*^th^ direction in a set. This was done for 512 rotations of **D**, which yielded 512 realizations of the powder-averaged signal, denoted $\bar{S}_{j}\left( b \right)$. The rotational variance was quantified in terms of the coefficient of variation (CV) of the powder-averaged signal across all rotations. The CV was computed for *n*_dir_ between 1 and 64. Each set of directions was independently optimized through electrostatic repulsion on the half-sphere [14, 15] where the optimization was repeated 10^3^ times with random initialization to avoid sub-optimal direction sets [16]. The threshold for rotation invariance was set to CV < 1%. The minimum number of directions necessary to meet this condition (*n*_min_) was calculated as a function of FA ∈ [0, 1] and *b*·MD ∈ [0, 4]. The necessary number of directions is presented in S1 Fig. The figure shows that linear encoding in coherent white matter at *b* = 2 ms/µm^2^ (*b*·MD ≈ 2 and FA ≈ 0.9) requires *n*_min_ = 16. For low b-values, *n*_min_ approaches 1, however, in practice we used a minimum of at least 6 directions for each b-value to reduce the influence from outliers. Note that a minimal protocol must include three b-values, each configured to render a rotation-invariant powder average. If STE can be assumed to be rotation invariant, the ‘very minimal’ protocol in white matter could comprise as little as one b = 0 sample, 26 LTE samples at *b* = 1 and 2 ms/µm^2^, and two STE samples at the same non-zero b-values; a total of 29 signal samples. Such protocols support model inversion but would likely exhibit marked parameter bias for low SNR and a high parameter variance. Although the optimal protocol remains an open question, the protocol suggested herein provides a tradeoff between acquisition time, accuracy and parameter precision that can be acquired in less than 10 minutes for all investigated configurations.


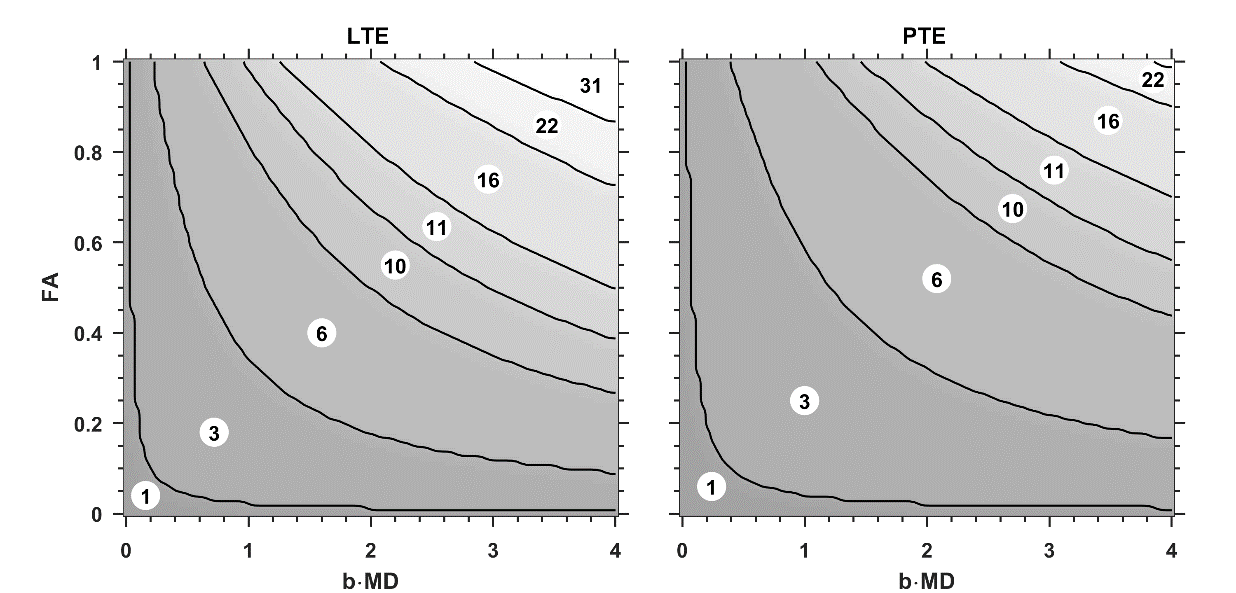


**S1 Fig. Minimal number of directions (***n*_min_**) required to yield a rotation invariant powder-averaged signal** for linear and planar tensor encoding (LTE and PTE). *n*_min_ can be read from the figure for combinations of the tissue fractional anisotropy (FA) and attenuation factor (*b*·MD), where the numbers in the circles show *n*_min_ for a color-coded interval. As expected, higher anisotropy and attenuation both demand a larger number of diffusion-encoding directions. Spherical tensor encoding always requires *n*_min_ = 1, since it is inherently invariant to rotation. Note that we consider the signal to be rotation invariant when CV < 1%. The PTE plot is included for completeness, even if it was not used in the data acquisition of this study.

# References

1. Jensen JH, Helpern JA. MRI quantification of non-Gaussian water diffusion by kurtosis analysis. NMR Biomed. 2010;23(7):698-710. doi: 10.1002/nbm.1518. PubMed PMID: 20632416; PubMed Central PMCID: PMC2997680.

2. Hui ES, Jensen JH. Double-pulsed diffusional kurtosis imaging for the in vivo assessment of human brain microstructure. Neuroimage. 2015;120:371-81. doi: 10.1016/j.neuroimage.2015.07.013. PubMed PMID: 26172309.

3. Topgaard D, Söderman O. Experimental determination of pore shape and size using q-space NMR microscopy in the long diffusion-time limit. Magn Reson Imaging. 2003;21(1):69-76. doi: 10.1016/s0730-725x(02)00626-4.

4. Le Bihan D, Breton E, Lallemand D, Grenier P, Cabanis E, Laval-Jeantet M. MR imaging of intravoxel incoherent motions: application to diffusion and perfusion in neurologic disorders. Radiology. 1986;161(2):401-7. doi: 10.1148/radiology.161.2.3763909. PubMed PMID: 3763909.

5. Szczepankiewicz F. Imaging diffusional variance by MRI: The role of tensor-valued diffusion encoding and tissue heterogeneity: Lund University; 2016.

6. Wong EC, Cox RW, Song AW. Optimized isotropic diffusion weighting. Magn Reson Med. 1995;34(2):139-43. PubMed PMID: 7476070.

7. Mori S, van Zijl P. Diffusion Weighting by the Trace of the Diffusion Tensor within a Single Scan. Magn Reson Med. 1995;33(1):41-52. doi: DOI 10.1002/mrm.1910330107. PubMed PMID: WOS:A1995PZ80800006.

8. Moffat BA, Chenevert TL, Lawrence TS, Meyer CR, Johnson TD, Dong Q, et al. Functional diffusion map: a noninvasive MRI biomarker for early stratification of clinical brain tumor response. Proc Natl Acad Sci U S A. 2005;102(15):5524-9. doi: 10.1073/pnas.0501532102. PubMed PMID: 15805192; PubMed Central PMCID: PMCPMC555936.

9. Sjölund J, Szczepankiewicz F, Nilsson M, Topgaard D, Westin CF, Knutsson H. Constrained optimization of gradient waveforms for generalized diffusion encoding. J Magn Reson. 2015;261:157-68. doi: 10.1016/j.jmr.2015.10.012. PubMed PMID: 26583528.

10. Szczepankiewicz F, Nilsson M, editors. Maxwell-compensated waveform design for asymmetric diffusion encoding. Proc Intl Soc Mag Reson Med 26; 2018; Paris, France.

11. Topgaard D. NMR methods for studying microscopic diffusion anisotropy. In: Valiullin R, editor. Diffusion NMR in Confined Systems: Fluid Transport in Porous Solids and Heterogeneous Materials. New Developments in NMR: Royal Society of Chemistry, Cambridge, UK; 2016.

12. Edén M. Computer simulations in solid-state NMR. III. Powder averaging. Concepts in Magnetic Resonance Part A. 2003;18A(1):24-55. doi: 10.1002/cmr.a.10065.

13. Szczepankiewicz F, Westin CF, Ståhlberg F, Lätt J, Nilsson M, editors. Minimum number of diffusion encoding directions required to yield a rotationally invariant powder average signal in single and double diffusion encoding. Proc Intl Soc Mag Reson Med 24; 2016; Singapore.

14. Jones DK, Horsfield MA, Simmons A. Optimal strategies for measuring diffusion in anisotropic systems by magnetic resonance imaging. Magn Reson Med. 1999;42(3):515-25. Epub 1999/09/01. PubMed PMID: 10467296.

15. Leemans A, editor ExploreDTI: a graphical toolbox for processing, analyzing, and visualizing diffusion MR data. Proc Intl Soc Mag Reson Med; 2009.

16. Cook PA, Symms M, Boulby PA, Alexander DC. Optimal acquisition orders of diffusion-weighted MRI measurements. J Magn Reson Imaging. 2007;25(5):1051-8. doi: 10.1002/jmri.20905. PubMed PMID: 17457801.
